# Efficacy of embolotherapy for the treatment of pelvic congestion syndrome: A systematic review

**DOI:** 10.1007/s11845-024-03608-6

**Published:** 2024-01-31

**Authors:** Joseph Hanna, Joshua Bruinsma, Hugo C. Temperley, Dhanushke Fernando, Niall O’Sullivan, Mark Hanna, Ian Brennan, Stefan Ponosh

**Affiliations:** 1 Department of Surgery, St. John of God Subiaco, Perth, WA Australia; 2 Department of Surgery, St. James’s Hospital, Dublin, Ireland; 3 Department of Radiology, St. James’s Hospital, Dublin, Ireland; 4 Ponosh Vascular, Hollywood Consulting Centre, Perth, WA Australia

**Keywords:** Embolisation, Interventional radiology, Pelvic congestion syndrome, Quality of life

## Abstract

Pelvic congestion syndrome (PCS) poses a significant health, diagnostic, and economic challenges. Transcatheter embolisation has emerged as a promising treatment for PCS. A systematic review was performed in order to assess the safety and efficacy of transcatheter embolisation in the treatment of PCS. A systematic search of electronic databases was performed using ‘PubMed’, ‘Embase’, ‘Medline (OVID)’, and ‘Web of Science’, for articles pertaining to efficacy of embolotherapy for the treatment of pelvic congestion syndrome. A total of 25 studies were included in this systematic review with a combined total of 2038 patients. All patients included were female with a mean average age of 37.65 (31–51). Of the 25 studies, 18/25 studies reported pre- and post-procedural pelvic pain outcomes using a visual analogue scale (VAS). All studies showed a reduction in VAS post-procedure. Transcatheter embolisation had a high technical success rate (94%) and an overall complication rate of 9.0%, of which 10.4% were major and 89.6% were minor. Fifteen out of 19 (78.9%) major complications required a subsequent intervention. Transcatheter embolisation using various techniques is effective and safe in treating PCS. A low quality of evidence limits the currently available literature; however, embolisation has shown to improve symptoms in the majority of patients with low complication rates and recurrence rates.

**Supplementary Information:**

The online version contains supplementary material available at 10.1007/s11845-024-03608-6.

## Background

Chronic pelvic pain (CPP) is a common condition affecting up to a quarter of the female population worldwide [1]. It is characterised by pain originating from the pelvis that lasts more than 6 months and is associated with negative cognitive, behavioural, sexual and emotional consequences [12]. CPP accounts for up to 40% of gynaecological laparoscopies and approximately 20% of all gynaecology outpatient appointments, creating a significant economic burden on health systems [3, 4].

Thirty to 40% of cases of CPP are associated with pelvic congestion syndrome (PCS) [7]. PCS consists of a number of clinical symptoms associated with concomitant anatomical abnormalities secondary to pelvic venous insufficiency, usually reflux of the ovarian or internal iliac veins [2]. The aetiology of PCS is not completely understood but is hypothesised to involve hormonal, mechanical, and vasoactive factors leading to venous dilatation (>5 mm), insufficiency, and pelvic venous reflux [3]. Patients with PCS often present with non-cyclical chronic pelvic pain worsened by standing, associated with dyspareunia or lower urinary tract symptoms, and may have perineal, buttock, vulval, or vaginal varicosities on examination [4].

Several therapeutic options have been shown to successfully alleviate pain in patients suffering from PCS including medical, surgical, and endovascular therapies. Medical therapies range from simple analgesia to hormonal therapies (medroxyprogesterone acetate/gonadotropin receptor agonists) or venoactive therapies (micronised purified flavonoid fraction). Surgical options include open/laparoscopic ovarian vein embolisation or hysterectomy with salpingo-oophorectomy. Transcatheter embolisation has emerged as one of the effective treatments for PCS, with technical success rate of 98-100% and symptom improvement at 1-5 years of follow-up in 80-93% of patients [5–9]. This technique aims to occlude venous axes that have been proven to be insufficient with venography performed in the same procedure, and can be safely performed in an ambulatory outpatient clinic as a day procedure [10, 11].

PCS poses significant health, diagnostic, and economic challenges. A systematic review was performed in order to assess the efficacy and safety of transcatheter embolisation in the treatment of PCS.

## Methods

### Search strategy and data extraction

**Fig. 1:**
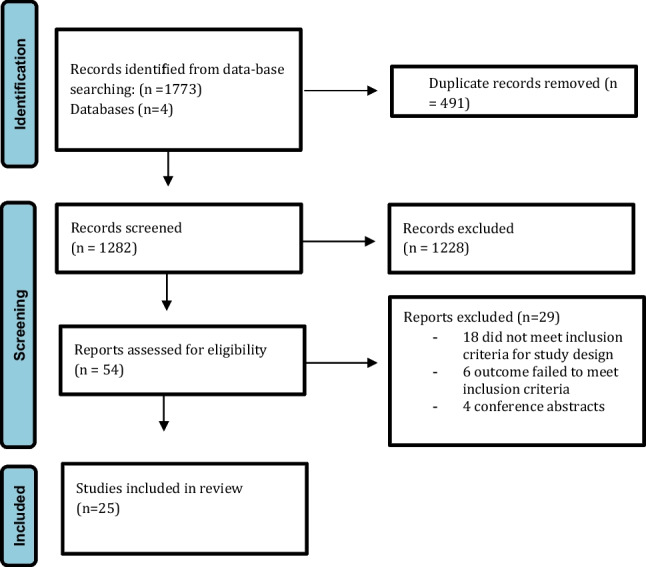
PRISMA flowchart outlining the systematic search process

A systematic review was conducted in accordance to the *Preferred Reporting Items for Systematic Reviews and Meta-Analyses* (PRISMA) guidelines. A formal systematic search was performed of the PubMed, Embase, Medline (OVID), and Web of Science databases using keywords and MeSH terms to identify relevant titles up to and including August 21st, 2022, for studies relating to the efficacy of embolotherapy for the treatment of pelvic congestion syndrome (Fig. 1). Grey literature and reference lists of relevant articles were reviewed for relevant studies. This study was prospectively registered with the International Prospective Register of Systematic Reviews in the PROSPERO platform with the reference number CRD42022374727.

The search was performed by two independent reviewers (JH and HT), using a predetermined search strategy that was designed by the senior authors. Details in relation to the search strategy can be found in Supplementary Appendix 1 (S1). Retrieved studies were reviewed to ensure studies met the predefined inclusion and exclusion criteria (see below). Discordances in opinion were resolved through consultation with a third author (NOS). Manual cross-referencing of reference lists from studies was undertaken to identify further potential articles for inclusion.

Two reviewers, JH and HT, independently extracted data from the studies identified. They recorded study details, basic patient clinicopathological characteristics, management strategies, and outcomes. The information was extracted based on the PICOTS framework, which includes population, intervention, comparator, outcomes, timing, and setting. GraphPad Prism (version 8.3.0) was used to analyse and create figures.

### Eligibility criteria

The inclusion criteria were as follows: (1) published studies demonstrating the efficacy of embolotherapy for the treatment of pelvic congestion syndrome, (2) published in the English language, and (3) published after 1995.

The exclusion criteria were as follows: (1) abstract only, (2) studies failing to discuss or denote PCS, (3) paediatric cohorts aged less than 18 years, and (4) case reports.

### Risk of bias

Assessment of potential biases within included RCTs was assessed using the Cochrane Collaboration (for randomised controlled trials (RCTs)) [12, 13]. This assessment tool grades each study as being high (red circle), low (green circle), or unclear (yellow) risk of bias across six categories. For non-RCT studies, Newcastle–Ottawa scale (NOS) risk of bias tool (2) and the results were tabulated. This assessment tool grades each study as being ‘satisfactory’ or ‘unsatisfactory’ across various categories. We assigned stars to evaluate study quality: 7 stars, ‘very good’; 5–6 stars ‘good’; 3–4 stars, ‘satisfactory’; and 0–2 stars, ‘unsatisfactory’. The critical appraisal was completed by two reviewers independently (JH and HT), where once again a third reviewer (NOS) was asked to arbitrate in cases of discrepancies in opinion.

### Aims and objectives

The overall aims and objectives of this study were as follows:

- Primary outcome:
  1. Improvement in pelvic pain measured subjectively or objectively using a 0 to 10 visual analogue score (VAS)
- Secondary outcomes:
  1. Other PCS symptom improvement measured subjectively or objectively using a 0 to 10 VAS, including dysmenorrhoea, lower limb pain, and dyspareunia
  2. Reintervention rate, which was defined as any subsequent intervention due to a sequelae of the patient’s symptoms
  3. Recurrence rate, which was defined as radiological or symptomatic recurrence of initial symptoms after index treatment.
  4. Complication rate, classified according to the Society of Interventional Radiology (SIR) classification system for complications by outcome [14]:
    - Post-embolisation syndrome, defined as the occurrence of gluteal and lumbar post-procedural pain, and/or abdominal pain in the area of the vessel embolised and/or pyrexia [15].
    - Access complications, defined as the presence of a haematoma at the procedural puncture site [16].
    - Coil migration, defined as entire or free fragment of coil deployed in a normal vessel, or any vessel other than the target vessel [17].
    - Reintervention, defined as a subsequent procedure required due to a complication from the index procedure
  5. Technical success, defined as successful occlusion of the target vein demonstrated to display reflux on venography or pre-operative imaging

## Results

### Study selection/included studies

The systematic search strategy identified a total of 1773 studies, of which 491 duplicate studies were manually removed. The remaining 1282 studies were screened for relevance, before 54 full texts were reviewed. In total, 25 studies fulfilled our inclusion criteria and were included in this systematic review (Fig. 1) [6–9, 18–38]. Due to heterogeneity in study method and results presented, a meta-analysis was not performed, and a collative summary of findings was deemed appropriate.

### Baseline characteristics

The data collected were highly heterogeneous and descriptive. The majority of studies had similar and consistent study population groups, with all 25 studies focusing on females undergoing pelvic venous embolisation for pelvic congestion syndrome. The study design was a retrospective cohort study in 13/25 reviews [8, 19, 23–25, 27, 29, 32–36], prospective cohort study in 8/25 reviews [7, 18, 20–22, 26, 28, 37, 38], and case series (with greater than 10 patients in 2/25 reviews) [30, 31], and 2/25 studies were randomised clinical trials (RCT) [6, 9]. The combined total of patients was 2038 across all 25 studies (range in sample size, 11–520). The mean average age 37.65 (range of reported means, 31–51). All patients were female, with no reported male cases. Of the 25 studies included in this analysis, the three most common countries of publication were Spain (5/25) [7, 9, 18, 19, 32], France (5/25) [8, 21, 23, 38], and the USA (3/25) [28, 31, 36]. The mean follow-up time was 23.54 months (range, 4.5–60). Publication dates ranged from 1997 to 2022. The primary outcome was improvement in VAS for CPP in 18/25 studies [6–8, 18–20, 23, 24, 26, 28, 29, 32–38]. A summary of study characteristics is presented in Table 1.

**Table 1:** Study characteristics

**Author**	**Year**	**Country**	**Study period**	**Study design**	**Study group**	**Method of embolisation**	**Sample size**	**Primary outcomes reported**	**Secondary outcomes reported**	**Follow-up period**
Asciutto et al. [29]	2009	Germany	2001–2004	Retrospective review	Females with pelvic reflux undergoing pelvic venous embolisation	Coils	35	VAS for CPP	N/A	Mean 45 months
Capasso et al. [30]	1997	Belgium	1993–1995	Case series	Females with pelvic reflux undergoing pelvic venous embolisation	Sclerotherapy ± coils	19	Subjective improvement	Other symptoms: dyspareunia	Mean 15.4 months (range 10–32)
Chung and Huh [6]	2003	Korea	1998–2002	RCT	Female patients with PCS undergoing pelvic venous embolisation	Coils	118	VAS for CPP	Stress levels	Mean 26.6 (± 5.2)
Cordts et al. [31]	1998	USA	N/A	Case series	Female patients with PCS undergoing pelvic venous embolisation	Coils	11	% improvement in symptoms	N/A	Mean 13.4 months (3–28)
De Gregorio et al. [32]	2020	Spain	2000–2017	Retrospective review	Female patients with pelvic reflux undergoing pelvic venous embolisation	Coils or Amplatzer plugs	520	VAS for CPP	Recurrence of symptoms Recurrence of lower limb varices	Mean 58.7 months (± 5.7) (36–60)
Gandini et al. [33]	2014	Italy	2005–2011	Retrospective review	Female patients having embolisation for PCS with high-outflow venous collaterals	Foam	26	VAS for CPP	Other symptoms: urinary urgency, dyspareunia, dysmenorrhoea, recurrence at 12 months	12 months
Gavrilov et al. [34]	2021	Russia	2012–2019	Retrospective review	Female patients with PCS undergoing treatment	Coils	95	VAS for CPP	Other symptoms: daily activity score, social activity score, disability days	36 months
Gong et al. [35]	2021	China	2013–2020	Retrospective review	Female patients diagnosed with pelvic venous reflux undergoing ovarian vein embolisation	Glubran-2	21	VAS for CPP	Nil	12 months
Guirola et al. [9]	2018	Spain	2014–2015	RCT	Females with pelvic venous insufficiency on TVUS	Coils vs. Vascular Plugs (Amplatz)	100	VAS for CPP	Other symptoms: dyspareunia, dysmenorrhoea, urinary urgency	12 months
Hocquelet et al. [23]	2014	France	2008–2012	Retrospective review	Females undergoing pelvic venous embolisation	Sclerotherapy and coils	33	VAS for CPP	Patient opinion on treatment Other symptoms: dyspareunia and dysuria	Mean 26 months (Range 3–59 months)
Jambon et al. [38]	2022	France	2017–2019	Prospective	Females undergoing pelvic venous embolisation	Onyx	73	VAS global symptoms	Other symptoms: pelvic pain, dyspareunia, post-coital pain, menstrual pain, LL pain, aesthetic discomfort, impact on daily life and working life	Median 28 months (Range 18.1–34.5)
Kim et al. [36]	2006	USA	1998–2003	Retrospective analysis	Females with CPP undergoing embolotherapy	Foam and coils	127	VAS for CPP	Other symptoms: overall pain, pain on standing, pain on lying down, dyspareunia, urinary frequency, menstrual pain, pain medications	Mean 45 months (± 18)
Laborda et al. [7]	2013	Spain	2001–2007	Prospective	Females undergoing pelvic venous embolisation	Coils	202	VAS for CPP	Recurrence of LL varices	60 months (1, 3, 6 months, yearly thereafter for 5 years)
Lorenzo et al. [18]	2022	Spain	2017–2019	Prospective	Females with PCS and bilateral ovarian vein reflux	Ethiodised oil and N2BCA	30	Pain on standing, dyspareunia, menstrual pain	N/A	Mean 24.5 months (± 6.5) (3, 6, 12 months, yearly thereafter)
Maleux et al. [22]	2000	Belgium	1993–1998	Prospective	Females with CPP and OV reflux on venography	Ethiodised oil	41	Clinical efficacy	N/A	Mean 19.9 months (range 1–61 months)
Marcelin et al. [21]	2017	France	2012–2016	Prospective	Females with PCS Diagnosed clinically and radiologically	Onyx	17	Resolution of symptoms	N/A	Mean 24.2 months (range 6–69 months)
Meneses et al. [37]	2013	Chile	Not stated	Prospective	Women with diagnosis of PCS with recurrent LL varices	Sclerotherapy and coils	10	VAS for CPP	Recurrence of VV	3, 6 months
Nasser et al. [19]	2014	Spain	2001–2011	Retrospective review	Females with PCS undergoing endovascular embolisation of ovarian & pelvic varicose veins	Coils	113	VAS for CPP	Other symptoms: dyspareunia, dysmenorrhoea, urinary symptoms, and LL symptoms	12 months (1, 3, 6, 12 months)
Pyra et al. [20]	2016	Poland	2014	Prospective	Females with PCS undergoing embolisation using ArtVentine EOS. PCS diagnosed by TV doppler or pelvic MRI	Ethiodised oil	12	VAS for CPP	N/A	6 months
Senechal et al. [8]	2021	France	2014–2019	Retrospective review	Females with CPP and varicose veins of lower limbs undergoing pelvic venous embolisation	Onyx	327	VAS for CPP	Other symptoms: dyspareunia, micturitional urgency QoL	Mean 39 months (± 18)
Siqueira et al. [24]	2016	Brazil	2011–2015	Retrospective review	Females with PCS undergoing pelvic venous embolisation	Coils	22	VAS for CPP	N/A	Mean 10.2 months (± 7.9)
Tinelli et al. [25]	2012	Italy	2006–2010	Retrospective review	Females with pelvic reflux on imaging	Sclerotherapy (atossisclerol 3%)	28	VAS for CPP	N/A	6 months (10, 30, and 180 days)
Tropeano et al. [26]	2008	Italy	2005	Prospective	Females with CPP, negative lap, pelvic reflux on imaging	Sclerotherapy (atossisclerol 3%)	20	VAS for CPP	Recurrence on ultrasound	12 months (3, 6, and 12 months)
Van der Vleuten et al. [27]	2012	Netherlands	2003–2008	Retrospective review	Females with PCS undergoing pelvic venous embolisation	Ethiodised oil	21	Pain	N/A	Mean 18.1 months (± 11.6)
Venbrux et al. [28]	2002	USA	1998–2000	Prospective	Females with CPP due to pelvic reflux	Sclerotherapy and coils	56	Pain	Menstrual cycle changes	Mean 22.1 months

*LL* lower limb, *VAS* visual analogue scale, *CPP* chronic pelvic pain, *QoL* quality of life, *VV* varicose veins, ± standard deviation

### Risk of bias

Both of the included RCTs were ‘low risk’ of bias for most of the categories, using the Cochrane Collaboration risk of bias assessment for RCTs. The RCTs failed to provide detailed information in relation to their blinding process, rendering them ‘intermediate risk’ of bias. In regard to non-RCT studies, one study was ‘very good’, fourteen studies were ‘good’, seven studies were ‘satisfactory’, and one study was ‘unsatisfactory’. S1&2 summarises the results of our risk of bias assessment.

### Intervention technique

The technique used for embolisation varied between studies. All 25 studies reported the access vessel(s) used. The most common access vessel utilised for vessel puncture was the left or right common femoral vein, which was accessed in 21 studies [6–8, 19–22, 24–36, 38]. All 25 studies reported the target vein embolised, of which there were 5126 veins embolised in 2038 patients. As seen in Table 2, the most common target vein was the left ovarian vein (LOV). Fluoroscopy time was reported in 8/25 studies, with a mean time of 33.3 min (SD 18.7, range 7.6–43.3) [7, 9, 18, 23, 25, 30, 32, 33]. The embolotherapy technique was reported in all studies. The most common embolotherapy was the use of coils only which was used in 9/25 studies [6, 7, 9, 19, 24, 29, 31, 32, 34]. PVE was predominantly performed by interventional radiology in 21/25 (84%) studies [7–9, 18–28, 30, 32, 33, 35–38], followed by vascular surgery in 3/25 (12%) studies [29, 31, 34].

**Table 2:** Intervention details

**Intervention details**	***N***
Access vein	
* Common femoral vein*	*21*
* Brachial vein*	*4*
* Jugular vein*	*4*
* Basilic vein*	*1*
Target vein embolised	
* LOV*	1974
* ROV*	1127
* LIIV*	857
* RIIV*	770
* L hypogastric vein*	184
* L obturator vein*	98
* R obturator vein*	114
Embolotherapy type	
* Coils only*	9
* Sclerotherapy*	7
* Coils and sclerotherapy*	5
* Onyx*	3
* Vascular plugs*	3
Interventionalist	
* Interventional radiology* * Vascular surgery* * Not specified*	21 3 1

*LOV* left ovarian vein, *ROV* right ovarian vein, *LIIV* left internal iliac vein, *RIIV* right internal iliac vein

### Primary outcome

Overall, 18/25 studies reported pre- and post-procedural pain outcomes using a visual analogue scale (VAS) [6–8, 18–20, 23, 24, 26, 28, 29, 32–38]. As indicated in Table 3, all but one showed statistically significant reduction in VAS post-procedure. Pyra et al. did not report a *p*-value but demonstrated a reduction in VAS from 7.3 pre-procedure to 1.6 post-procedure [20]. Furthermore, 17/25 studies reported a qualitative improvement in pain. These findings are summarised in Table 4 [7–9, 19, 21–24, 26–31, 34–36]. Of note, 5/17 studies showed a proportion of patient who’s symptoms worsened at the time of follow-up [7, 8, 27, 29, 36].

**Table 3:** VAS improvement pre- and post-procedure

**Studies**	**VAS before**	**VAS after**	**Mean follow-up time (months)**	***p*-value**
**Chronic pelvic pain**				
Laborda et al. [7]	7.3	0.8	60	< 0.001
Lorenzo et al. [18]	7.7	2.2	12	< 0.001
Nasser et al. [19]	7.3	0.5	12	< 0.001
Pyra et al. [20]	7.3	1.6	6	NR
Hocquelet et al. [23]	7.4	1.4	26	< 0.001
Senechal et al. [8]	7.0	1.2	12	< 0.001
Siqueira et al. [24]	8.4	5.2	10	NR
Tropeano et al. [26]	8.0	3.0	12	< 0.001
Venbrux et al. [28]	7.8	2.7	12	< 0.025
Asciutto et al. [29]	5.2	1.2	36	< 0.0001
Chung and Huh [6]	7.8	3.2	12	< 0.001
De Gregorio et al. [32]	7.6	0.9	59	0.0016
Gandini et al. [33]	7.5	2.8	12	< 0.01
Gavrilov et al. [15]	7.5	0.5	6	< 0.05
Gong et al. [35]	7.6	0.4	12	< 0.05
Jambon et al. [38]	6.1	1.4	3	< 0.05
Kim et al. [36]	7.6	2.9	45	< 0.0001
Meneses et al. [37]	8.2	4.0	3	< 0.01
**Dyspareunia**				
Lorenzo et al. [18]	7.22	2.55	12	0.008
Senechal et al. [8]	4.7	0.8	12	< 0.001
Gandini et al. [33]	6.1	1.7	12	< 0.05
Jambon et al. [38]	3.84	0.81	3	< 0.05
Kim et al. [36]	3.3	1.5	45	< 0.000001
**Dysmenorrhoea**				
Lorenzo et al. [18]	4.63	2.22	12	0.031
Gandini et al. [33]	5.3	1.6	12	< 0.05
Jambon et al. [38]	5.19	1.14	3	< 0.05
Kim et al. [36]	4.9	2.2	45	< 0.000001
**Lower leg pain**				
Senechal et al. [8]	4.4	1.8	12	< 0.001

*NR* not reported

**Table 4:** Qualitative improvement in CPP

**Study**	**Improvement** ***N* (%)**	**No improvement** ***N* (%)**	**Worsening** *N*** (%)**	**Sample size** ***N***
Laborda et al. [7]	188 (93.0%)	11 (5.5%)	3 (1.5%)	202
Nasser et al. [19]	113 (100%)	0 (0%)	0 (0%)	113
Marcelin et al. [21]	13 (76.5%)	4 (23.5%)	0 (0%)	17
Maleux et al. [22]	28 (68.3%)	13 (41.7%)	0 (0%)	41
Hocquelet et al. [23]	31 (93.9%)	2 (6.1%)	0 (0%)	33
Senechal et al. [8]	256 (88.9%)	29 (10.1%)	3 (1%)	288
Siqueira et al. [24]	17 (77.3%)	5 (22.7%)	0 (0%)	22
Tropeano et al. [26]	17 (85.0%)	3 (15.0%)	0 (0%)	20
Van der Vleuten et al. [27]	13 (61.9%)	7 (33.3%)	1 (4.8%)	21
Venbrux et al. [28]	56 (100%)	0 (0%)	0 (0%)	56
Asciutto et al. [29]	26 (47.3%)	20 (36.4%)	9 (16.4%)	55
Capasso et al. [30]	14 (73.7%)	5 (26.3%)	0 (0%)	19
Cordts et al. [31]	9 (100%)	0 (0%)	0 (0%)	9
Gavrilov et al. [15]	68 (95.8%)	3 (4.2%)	0 (0%)	71
Gong et al. [35]	21 (100%)	0 (0%)	0 (0%)	21
Guirola et al. [9]	100 (100%)	0 (0%)	0 (0%)	100
Kim et al. [36]	106 (83.5%)	17 (13.4%)	5 (3.9%)	127

### Secondary outcome

#### Other symptoms of PCS

Other commonly reported outcomes were improvement in other symptoms such as dyspareunia and dysmenorrhoea; these are summarised in Table 3. Five studies (5/25) reported pre- and post-procedure VAS scores for dyspareunia [8, 18, 33, 36, 38]. Pre- and post-procedure VAS scores for dysmenorrhoea were reported in 4/25 studies [18, 33, 36, 38]. Qualitative improvements were recorded for a variety of symptoms amongst the studies, including dyspareunia (8/25) [9, 18, 23, 30, 31, 33, 36, 38], dysmenorrhoea (6/25) [9, 18, 31, 33, 36, 38], lower limb pain (2/25) [8, 38], post-coital pain (3/25) [8, 31, 38], and urinary symptoms (5/25) [9, 23, 31, 33, 36].

#### Symptom recurrence and reintervention

Symptom recurrence during the follow-up period was reported in 17/25 studies and ranged from 0 to 42% during the follow-up period [8, 18, 21, 23–28, 30–34, 36, 37]. The most common methods of reintervention were repeat embolisation procedure and hysterectomy. These findings are summarised in Table 5.

**Table 5:** Recurrence and reintervention rate

**Author**	*Recurrence* *N *(%)	*Reintervention*	*Re-embolisation*	*Hysterectomy*
Laborda et al. [7]	24 (12%)	0	0	0
Marcelin et al. [21]	5 (29%)	4	4	0
Hocquelet et al. [23]	6 (18%)	6	6	0
Senechal et al. [8]	14 (5%)	0	0	0
Siqueira et al. [24]	1 5(%)	0	0	0
Tinelli et al. [25]	0 (0%)	0	0	0
Tropeano et al. [26]	5 (25%)	3	3	0
Van der Vleuten et al. [27]	9 (43%)	9	9	0
Venbrux et al. [28]	3 (5%)	2	2	0
Capasso et al. [30]	5 (26%)	5	5	3
Cordts et al. [31]	2 (18%)	0	0	0
De Gregorio et al. [32]	26 (5%)	17	17	0
Gandini et al. [33]	1 (4%)	1	0	0
Gavrilov et al. [15]	0 (0%)	0	0	0
Guirola et al. [9]	9 (9%)	9	9	0
Kim et al. [36]	7 (5%)	5	0	5
Meneses et al. [37]	0 (0%)	0	0	0

#### Technical success and complications

Technical success was reported in 20/25 studies and was achieved in 1583/1676 patients (94%). Complications were grouped according to the SIR classification system for complications by outcome as seen in Table 6 [14]. The most common complication was post-embolisation syndrome defined as mild abdominal pain immediately post-operatively which was self-resolving or treated with simple analgesia. There were 15 complications that required surgical or radiological intervention, including 13 migrated coils that required snaring, one common femoral artery injury that resulted in a false aneurysm and required embolisation, and one patient who developed salpingitis 1 month post-procedure requiring surgical intervention. There were no complications that led to permanent adverse sequelae, or death of a patient.

**Table 6:** Complications graded by the Society of Interventional Radiology (SIR) classification

**Complication**	***N***
Total	183
SIR A	
Vessel perforation	22
Tachyarrhythmia	1
Phlebitis	4
Vagal reaction	1
Dyspnoea	4
Panic attack	1
Coil migration not snared	15
Access site haematoma	26
SIR B	
Post-embolisation syndrome	77
Mild contrast allergy	10
Pyrexia of unknown origin	3
SIR C	
DVT requiring anticoagulation	2
Bradycardia requiring atropine	1
Allergic bronchospasm	1
Coil migration requiring snaring	13
CFA injury requiring embolisation	1
SIR D	
Salpingitis requiring operation	1
SIR E	0
SIR F	0

*DVT* deep vein thrombosis, *CFA* common femoral artery

## Discussion

This systematic review supports pelvic venous embolisation (PVE) as a safe and effective treatment modality for pelvic congestion syndrome (PCS), with all studies demonstrating improvement in symptoms in a majority of patients, and low complication and recurrence rates. This systematic review adds a contemporary analysis of the literature, incorporating several embolotherapy techniques such as various sclerotherapy agents, vascular plugs, and coils. Furthermore, by analysing VAS scores, this review provides a more uniform and objective way to quantify the efficacy of embolisation in the treatment in PCS.

In the symptom profile of PCS, chronic pelvic pain is the most commonly reported. All 25 studies analysed showed improvement in chronic pelvic pain in the majority of participants. There is currently a sparsity of validated quality of life tools available for CPP which has led to the VAS being widely used [39]. The VAS allows a more objective method to measure and compare pain scores and was used in 18 of the 25 studies. All 18 studies that reported VAS showed a significant improvement in pre-procedural vs. post-procedural scores for pelvic pain. The 7 studies that did not utilise VAS score demonstrated a subjective improvement in pain scores. In these studies, 67–100% of participants reported reduction in pelvic pain post treatment. This systematic review supports the previous literature which has demonstrated the efficacy of PVE in reducing chronic pelvic pain in PCS. Brown et al. also reported improvement in clinical symptoms in 68–100% of patients, although this review was performed in 2018, and only reported the results of 14 papers [2]. Sutanto et al. examined outcomes of isolated coil embolisation only and reported a 5.47 point improvement on the VAS scale at 2 years after PVE in a pooled analysis of eight studies and a qualitative improvement in pain in the majority (70–100%) of patients in four studies [40].

Although chronic pelvic pain is the most described symptom of PCS, it can present with a variety of other debilitating symptoms including dyspareunia and dysmenorrhoea [10]. Significant quantitative improvement in both dyspareunia and dysmenorrhoea was seen in studies reporting VAS scores for these symptoms, although pre-procedural VAS scores were generally lower for other symptoms compared to pelvic pain. Qualitative information collected measuring other symptoms such as lower limb pain, post-coital pain, and lower urinary tract symptoms (LUTS) also demonstrated high rates of subjective improvement.

PVE has become a widely accepted treatment modality for PCS as it is less invasive than open surgical or laparoscopic ligation techniques and has been shown to be safely performed in an ambulatory day clinic using local anaesthetic only [10, 11]. This was reflected in this review with low complication rates reported across studies. In total, 183 complications occurred across 2038 procedures. The majority of these complications (89%) were self-limiting and did not require pharmacological, radiological, or surgical treatment, including post-embolisation syndrome (PES) and access site haematomas. Of the 183 complications, 15 (8%) complications required surgical or radiological intervention. The most common of these was coil migration into the pulmonary vasculature requiring snaring, usually performed at the time of the index procedure through the same access, without any deleterious long-term sequelae. Proportionally, our review found a higher number of major and minor complications compared to the review by Sutanto et al., who investigated coil embolisation only (9.0% vs. 3.8%). Although the addition of other embolisation techniques (such as foam sclerotherapy, ethiodised oil, and Onyx) into our systematic review seemingly increased the complication rates, closer analysis of the data shows a higher rate of non-PES complications in studies that used coil embolisation. Of note, the 8 studies with the highest rates of complications (8–45%) used coil embolisation [7, 9, 29–32, 34, 37], and the 5 studies that reported no complications used other embolotherapy techniques such as Onyx, foam sclerotherapy, ethiodised oil, and Glubran-2 [18, 21, 26, 27, 35]. However, given the heterogeneity of the studies, it is difficult to draw meaningful conclusions from this.

Symptom recurrence (7.4%) and reintervention rates (3.9%) were low, despite widely variable follow-up periods amongst ranging from 4.5 months to 5 years. These rates were comparatively low compared to previous studies, such as that of Sutanto et al. who reported a 10–30% reintervention rate, predominantly due to recurrent pelvic pain or recurrent pelvic varices [40]. There was no clear pattern with regard to an embolotherapy technique associated with lower recurrence or reintervention rates.

The literature comparing different embolotherapy techniques in the treatment of PCS is scarce which has led to significant heterogeneity in clinical practice. In our review, there were two studies that directly compared the outcomes of coil embolotherapy and vascular plugs for pelvic vein embolisation. Guirola et al. performed a randomised trial comparing the use of Amplatzer vascular plugs to fibred platinum coils and did not find any difference in improvement of VAS scores or complication rates between the two devices [9]. De Gregorio et al. retrospectively analysed patients undergoing pelvic venous embolisation with either Amplatzer vascular plugs or coils and similarly did not find any significant difference in short and long-term outcomes. Although current guidelines recommend pelvic venous embolisation as a safe treatment for patients with CPP due to PCS, there is no evidence-based recommendation for the optimal embolic material used, and this decision is largely physician dependent [41]. Further research comparing the various embolisation techniques are required.

There were several limitations of our review including the small number of studies with quantitative comparative data and the heterogeneity of their study designs, which precluded collative meta-analysis and large volume analysis. Furthermore, the majority of studies were retrospective in design and did not control for other confounding factors. Robust prospective, randomised controlled trials and qualitative Delphi studies are needed to define treatment and diagnostic protocols. Finally, the studies included in this study span 25 years (1997–2022) which brings into question the comparability of these data provided. Nonetheless, our study will impact clinical practice by allowing interventionalists and surgeons to counsel patients on the optimal management’s options and inform patient on expected outcomes.

## Conclusion

PCS is a prevalent condition that significantly impacts quality of life and poses a diagnostic and economic challenge to the public. According to current literature, PVE is a safe treatment option that effectively reduces symptom burden with relatively low complication rates. However, the studies reported in the literature were variable in sample size, follow-up period, and outcome description. Further research is required to draw conclusions about the longevity and optimal embolisation method, particularly regarding long-term follow-up and a head-to-head comparison of the embolotherapy technique.

### Supplementary Information

Below is the link to the electronic supplementary material.

Supplementary file1 (DOCX 19 KB)
