# Supplementary material for: Efficacy of embolotherapy for the treatment of pelvic congestion syndrome: A systematic review
Source: Ir J Med Sci. 2024 Jan 31;193(3):1441–51. doi: 10.1007/s11845-024-03608-6 (PMC11128397; doi:10.1007/s11845-024-03608-6)
Supplement: Supplementary file 1 — Supplementary file1 (DOCX 19 KB) [file 11845_2024_3608_MOESM1_ESM.docx]

**Supplementary appendix 1: Search Strategy**

The following databases were searched as part of the systematic review in August 2022: Medline (OVID),Embase, PubMed and Web of Science. The following search terms were used: “Pelvic Congestion syndrome*”, “Chronic pelvic pain*”, “pelvic*”, “congestion*”, “emboli*”, “embolisation*” and “embolization*”. The symbol “*” was used to allow variations on a word stem to be included in the search results. Furthermore, the following MeSH (medical subject headings) were used: (emboli[MeSH] OR Pelvic Congestion syndrome[MeSH]) AND (Emboli[MeSH] OR Chronic pelvic pain [MeSH]).The last date of search was 21 st August 2022. The grey literature was also searched to further identify ongoing works of literature. Additional articles were identified through searching the reference lists of relevant articles.

**Supplementary appendix 2: Risk of bias assessment (Cochrane collaboration risk of bias tool for RCTs)**

| **Author** | **Random sequence generation (SB)** | **Allocation concealment (SB)** | **Blinding of participants and**  **personnel (PB)** | **Blinding of outcome**  **assessment (DB)** | **Incomplete outcome data (AB)** | **Selective reporting (RB)** | **Other bias** |
| --- | --- | --- | --- | --- | --- | --- | --- |
| **Chung** | unclear | + | unclear | unclear | + | + | + |
| **Guirola** | unclear | unclear | unclear | unclear | + | + | + |

**Supplementary appendix 3: risk of bias assessment (Newcastle-Ottawa scale)**

| Author | **Selection** | | | | **Comparability** | **Outcome** | | | **Quality** |
| --- | --- | --- | --- | --- | --- | --- | --- | --- | --- |
|  | **Representativeness of the exposed cohort** | **Sample size (<25 = no star)** | **Non-respondents** | **Ascertainment of the exposure** | **The subjects in different outcome groups are comparable** | **Assessment of outcome** | **Statistical test** | **Period (<4weeks)** |  |
| **Laborda et al** | **🟑** | **🟑 ** | / | **🟑** | / | / | **🟑** | / | 4 |
| **Lorenzo et al** | **🟑** | **🟑 ** | / | **🟑** | / | **🟑** | **🟑** | **🟑** | 6 |
| **Nasser** | **🟑** | **🟑** | / | / | / | **🟑** | / | **🟑** | 4 |
| **Pyra et al** | **🟑** | **🟑 ** | / | / | / | / | / | / | 2 |
| **Scultetus** | **🟑** | **🟑** | **🟑** | / | / | / | **🟑** | **🟑** | 5 |
| **Marcelin** | **🟑** | **🟑** | **🟑** | / | / | / | **🟑** | **🟑** | 5 |
| **Maleux** | **🟑** | **🟑** | **🟑** | / | **🟑** | / | **🟑** | / | 5 |
| **Hocquelet** | **🟑** | **🟑** | / | / | / | / | **🟑** | / | 3 |
| **Senechal** | **🟑** | **🟑** | / | / | / | **🟑** | **🟑** | **🟑** | 5 |
| **Siqueiral** | **🟑** | **🟑** | **🟑** | **🟑** | / | **🟑** | **🟑** | **🟑** | 7 |
| **Tinelli** | **🟑** | / | / | / | / | / | **🟑** | / | 2 |
| **Tropeano** | **🟑** | / | **🟑** | / | **🟑** | / | / | **🟑** | 4 |
| **Van der Vleuten** | **🟑** | **🟑** | / | **🟑** | / | **🟑** | / | **🟑** | 5 |
| **Venbrux** | **🟑** | **🟑 ** | / | **🟑** | / | / | **🟑** | / | 4 |
| **Asciutto** | **🟑** | **🟑 ** | / | **🟑** | / | **🟑** | **🟑** | **🟑** | 6 |
| **Capasso** | **🟑** | **🟑** | / | / | / | **🟑** | / | **🟑** | 4 |
| **Cordts** | **🟑** | **🟑** | **🟑** | / | / | / | **🟑** | **🟑** | 5 |
| **De Gregorio** | **🟑** | **🟑** | **🟑** | / | / | / | **🟑** | **🟑** | 5 |
| **Gandini** | **🟑** | **🟑** | **🟑** | / | **🟑** | / | **🟑** | / | 5 |
| **Gavrilov** | **🟑** | **🟑** | / | / | / | / | **🟑** | / | 3 |
| **Hocquelet** | **🟑** | **🟑** | / | / | / | **🟑** | **🟑** | **🟑** | 5 |
| **Gong** | **🟑** | **🟑 ** | / | **🟑** | / | **🟑** | **🟑** | **🟑** | 6 |
| **Jambon** | **🟑** | **🟑** | **🟑** | / | / | / | **🟑** | **🟑** | 5 |
| **Kim** | **🟑** | **🟑** | **🟑** | / | / | / | **🟑** | **🟑** | 5 |
